# Comprehensive newborn screening for severe combined immunodeficiency, X-linked agammaglobulinemia, and spinal muscular atrophy: the Chinese experience

**DOI:** 10.1007/s12519-024-00846-7

**Published:** 2024-11-05

**Authors:** Chi Chen, Chao Zhang, Ding-Wen Wu, Bing-Yi Wang, Rui Xiao, Xiao-Lei Huang, Xin Yang, Zhi-Gang Gao, Ru-Lai Yang

**Affiliations:** 1 Department of Genetics and Metabolism, Children’s Hospital, Zhejiang University School of Medicine, National Clinical Research Center for Child Health, Hangzhou, China; 2 National Engineering Laboratory for Key Technology of Birth Defect Control and Prevention, Screening and Diagnostic R and D Center, Hangzhou, China

**Keywords:** Genetic diagnosis, Newborn screening, Severe combined immunodeficiency, Spinal muscular atrophy, X-linked agammaglobulinemia

## Abstract

**Background:**

Newborn screening (NBS) for severe combined immunodeficiency (SCID), X-linked agammaglobulinemia (XLA), and spinal muscular atrophy (SMA) enables early diagnosis and intervention, significantly improving patient outcomes. Advances in real-time polymerase chain reaction (PCR) technology have been instrumental in facilitating their inclusion in NBS programs.

**Methods:**

We employed multiplex real-time PCR to simultaneously detect T-cell receptor excision circles (TRECs), kappa-deleting recombination excision circles (KRECs), and the absence of the survival motor neuron (*SMN*) 1 gene in dried blood spots from 103,240 newborns in Zhejiang Province, China, between July 2021 and December 2022.

**Results:**

Of all the samples, 122 were requested further evaluation. After flow cytometry evaluation and/or genetic diagnostics, we identified one patient with SCID, two patients with XLA, nine patients with SMA [one of whom also had Wiskott–Aldrich Syndrome (WAS)], and eight patients with other medical conditions. The positive predictive values (PPVs) of NBS for SCID, XLA, and SMA were 2.44%, 2.78%, and 100%, respectively. The estimated prevalence rates in the Chinese population were 1 in 103,240 for SCID, 1 in 51,620 for XLA, and 1 in 11,471 for SMA.

**Conclusion:**

This study represents the first large-scale screening in mainland China using a TREC/KREC/SMN1 multiplex assay, providing valuable epidemiological data. Our findings suggest that this multiplex assay is an effective screening method for SCID, XLA, and SMA, potentially supporting the universal implementation of NBS programs across China.

**Graphical abstract:**

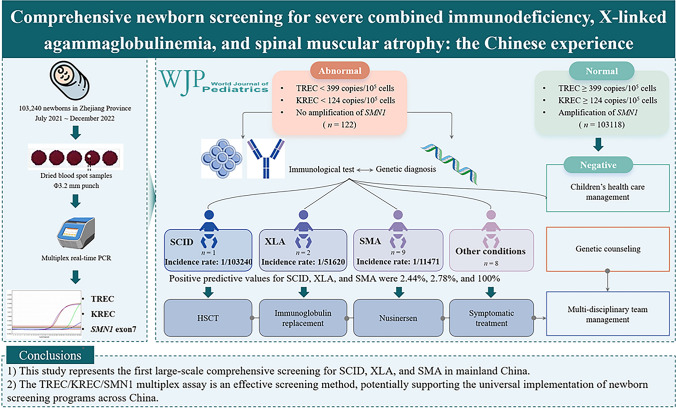

## Introduction

Newborn screening (NBS) aims to detect severe congenital and hereditary diseases in infants, facilitating early diagnosis and treatment to significantly improve the prognosis of affected patients [1]. Since the implementation of NBS for congenital hypothyroidism (CH) and phenylketonuria (PKU) in the 1980s, followed by congenital adrenal hyperplasia (CAH) and glucose-6-phosphate dehydrogenase (G6PD) deficiency in the 2000s, the application of tandem mass spectrometry (MS/MS) to over 40 genetic metabolic diseases has revolutionized the field of NBS [2]. Currently, NBS in China faces the challenge of expanding the range of target diseases that can be integrated into routine programs.

Severe combined immunodeficiency (SCID), one of the most severe forms of primary immunodeficiency disorders (PIDs), is caused by a spectrum of genetic mutations that result in critically impaired cellular and humoral immunity [3]. Real-time polymerase chain reaction (PCR)-based quantification of T-cell receptor excision circles (TRECs), which serve as DNA biomarkers of normal T-cell development, is widely used in NBS for SCID and can also identify other T-cell deficiencies [4, 5]. Patients diagnosed with SCID through NBS typically undergo hematopoietic stem cell transplantation (HSCT) at a significantly younger median age than those diagnosed based on clinical symptoms [6]. In general, patients with SCID who undergo HSCT at an earlier age experience superior outcomes compared to those who undergo transplantation later in life [7]. Kappa-deleting recombination excision circles (KRECs) are DNA fragments produced during B-cell maturation in the bone marrow [8]. Nakagawa et al. [9] described a PCR-based method for detecting KRECs, which was subsequently validated and utilized in pilot studies to identify B-cell deficiencies, such as X-linked agammaglobulinemia (XLA) [10–13]. The combined TREC/KREC assay provides a more comprehensive NBS approach for diverse forms of PIDs, enabling the detection of conditions that might be overlooked when using TRECs alone [14].

Spinal muscular atrophy (SMA) is a genetic disorder characterized by progressive, symmetrical muscle weakness and atrophy [15]. It is a common genetic condition that can lead to severe outcomes in infants, including death. SMA affects about 1 in 6000–11,000 live births, with an estimated carrier frequency of about 1 in 35–50 individuals [16]. SMA is primarily caused by mutations in the survival motor neuron (*SMN*) 1 gene located on chromosome 5q13, with about 95% of cases identified in newborns through screening for the homozygous absence of *SMN1* exon 7 [17]. The most profound therapeutic benefits of current treatments, such as onasemnogene abeparvovec [18], nusinersen [19], and risdiplam [20], are observed in patients who receive treatment prior to the manifestation of clinical symptoms. The American College of Medical Genetics and Genomics (ACMG) recommends population-wide screening for SMA, given the efficacy of these therapeutics and the additional benefits of early pre-symptomatic identification [21]. NBS for SMA using real-time PCR technology, similar to that employed in SCID-NBS, has been implemented in the United States of America (USA) and several other countries. In addition, determining the *SMN1* and *SMN2* copy numbers through multiplex ligation-dependent probe amplification (MLPA), the standard diagnostic method for SMA is crucial for clinical categorization and prognosis [22]. Previous studies have demonstrated the feasibility of using a multiplex qPCR assay to screen for both PID and SMA with the same real-time PCR technology [23, 24].

We conducted a study in Zhejiang Province to evaluate the effectiveness of incorporating TREC/KREC/*SMN1* screening into our NBS programs. This approach employs multiplex real-time PCR assays to screen newborns for severe PIDs by assessing T- and B-cell levels and detecting deletions in exon 7 of the *SMN1* gene. This study describes the NBS process and management of SCID, XLA, and SMA, based on the inaugural large-scale NBS implementation in the Mainland of China.

## Methods

### Dried blood spot samples

The NBS pilot trial for SMA, SCID, and XLA was conducted on newborns born in Zhejiang Province between July 2021 and December 2022. Dried blood spot (DBS) samples were collected 48 h after birth from adequately fed newborns. A 3.2-mm punch was extracted from the leftover DBS specimens collected during routine NBS at the Zhejiang Neonatal Screening Centre. The trial covered 303 maternity units across 75 counties in Zhejiang Province, excluding Ningbo City. This study was approved by the Research Ethics Committee of the Children’s Hospital of Zhejiang University School of Medicine (approval number: 2021-IRB-036) and compiled patient data without personal identifiers.

### TREC/KREC/*SMN1* screening assay

The TREC/KREC/*SMN1* NBS assay was performed using multiplex real-time PCR. DNA was extracted from 3.2-mm diameter punches (NeoMDx DNA Extraction kit, Xinbo, Suzhou, China). The DBS discs (3.2 mm) were punched and washed once in 100 μL of DNA elution buffer, then shaken at 1500 rpm for 10 min. After removing the wash buffer, 40 μL of fresh DNA elution buffer was added, and the samples were heated at 95 °C for 30 min. The eluted DNA from the supernatant underwent multiplex real-time PCR analysis to simultaneously quantify TREC, KREC, *SMN1* exon 7, and *RPP30* in 96-well formats using a NeoMDx PCR kit (Xinbo, Suzhou, China) on a SLAN® 96S real-time PCR instrument (Hongshi, Shanghai, China). The cycling conditions were as follows: 37 °C for 2 min, then 94 °C for 5 min, followed by 40 cycles of 93 °C for 10 s, 60 °C for 30 s, and 69 °C for 40 s. *RPP30* was used as an internal positive control for sample quantity. Individual cycle thresholds were determined by inspecting the amplification curves and automatically set using the instrument software. The quantities of TREC and KREC were calculated and expressed as copies per 10^5^ nucleated cells using the following formula:

$${\text{TREC}} \left( {{\text{KREC}}} \right){\text{copies}}/10^{5} {\text{cells}} = \frac{{{\text{Copy}} {\text{ number}} {\text{ of}} {\text{ TREC}} \left( {{\text{KREC}}} \right)}}{{\left( {{\text{Copy}} {\text{ number}} {\text{ of RPP}}30/2} \right)}} \times 10^{5}$$

Based on the distribution data of 3400 normal newborns, the optimal cutoff values for TREC and KREC copies/10^5^ cells were determined using the Youden index method, which was 0.005. The K**–**S test for these samples yielded a *P* value < 0.05, indicating that the distribution was almost normal. The cutoff values for TREC and KREC were set at < 399 copies/10^5^ cells and < 124 copies/10^5^ cells, respectively. Samples were classified as having a homozygous deletion of exon 7 of *SMN1* if no amplification of *SMN1* was detected. For TREC and KREC values, abnormal values were defined as those below the established cutoffs for TREC and/or KREC, provided there was no DNA amplification failure. All abnormal values and incomplete samples were confirmed using a repeat assay.

### Clinical procedures

After obtaining informed consent, referred infants were examined to confirm the diagnosis. A complete blood count, flow cytometry, and immunoglobulin levels were assessed for infants with suspected PID. Infants with critically low TREC and/or KREC values, particularly those with a suspected family history of immunodeficiency, underwent trio-whole exome sequencing (trio-WES) for comprehensive genetic analysis. To obtain more accurate epidemiological data and reduce the risk of missed diagnoses, referred infants with moderately low TREC and/or KREC values underwent whole exome sequencing (WES) as probands using genomic DNA extracted from fresh whole blood. Unreferred infants with abnormal TREC and/or KREC values also underwent WES, with genomic DNA extracted from their DBS. To minimize the inherent limitations of whole exome variant detection, the average sequencing depth was ×150, with more than 98% of the exonic regions achieving a coverage of over ×50, and 99% achieving a coverage of at least ×30. The probe detection method was used to capture the exonic and flanking intronic regions of the genome, and the obtained DNA sequences were compared to the human genome hg19 reference sequence from the University of California, Los Angeles (UCLA) database. Variants with at least tenfold coverage were analyzed using bioinformatic tools for pathogenicity, following data interpretation guidelines provided by the ACMG. Variant nomenclature was based on the guidelines set by the Human Genome Variation Society (HGVS). Only variants that fit the inheritance model of the patient’s family and were potentially linked to the fetal phenotypes were reported. All suspected clinically significant variants were verified using Sanger sequencing. Copy number variations (CNVs) were identified using the sliding window method based on read depth algorithms with WES data. MLPA was performed using whole blood samples for patients suspected of having SMA.

## Results

### Overall screening results

**Fig. 1:**
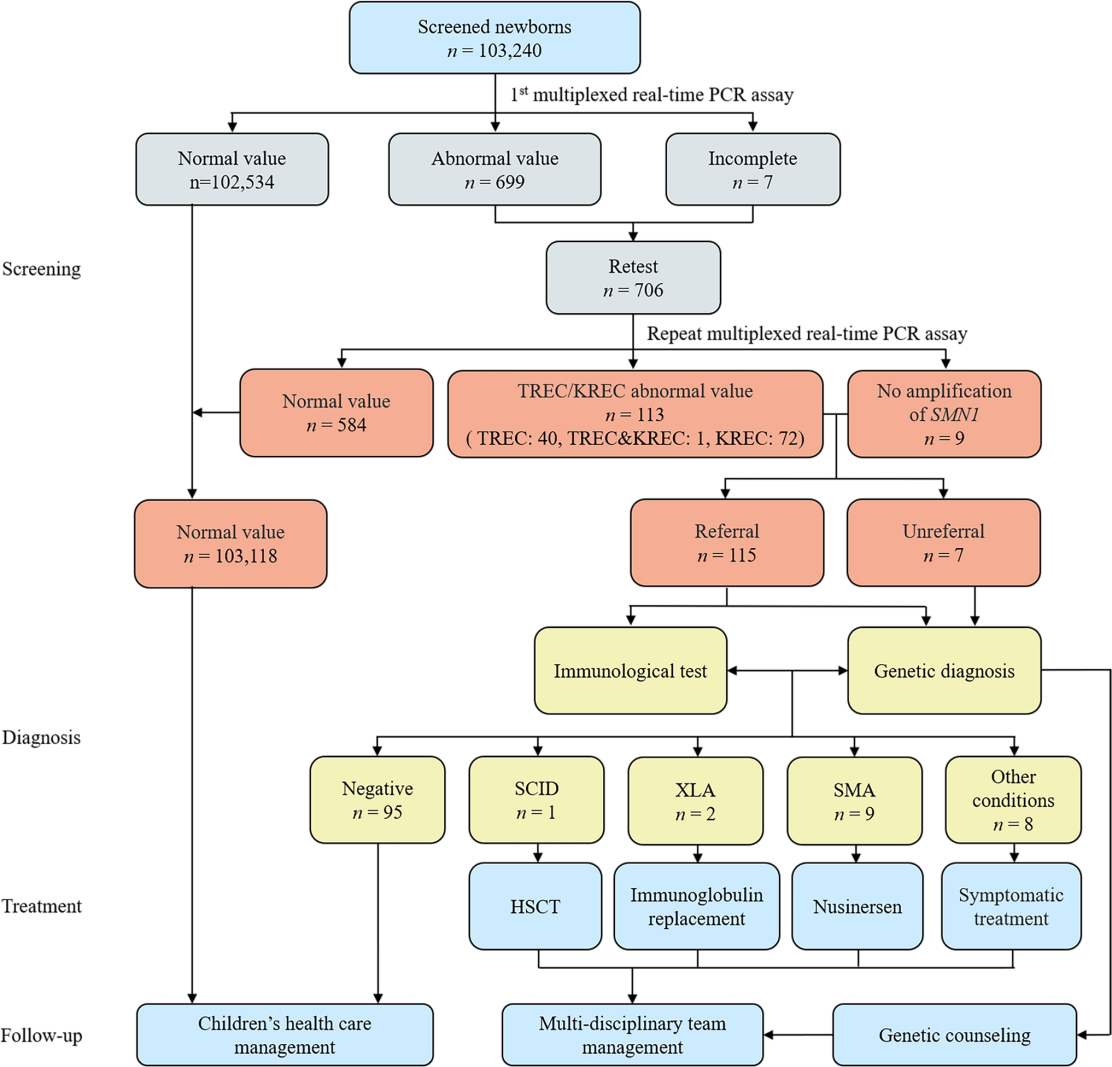
Schematic diagram of the T-cell receptor excision circle (TREC)/kappa-deleting recombination excision circle (KREC)/survival motor neuron 1 (*SMN1*) newborn screening, diagnostic, and treatment integration system. *PCR* polymerase chain reaction, *SCID* severe combined immunodeficiency, *XLA* X-linked agammaglobulinemia, *SMA* spinal muscular atrophy, *HSCT* hematopoietic stem cell transplantation

**Fig. 2:**
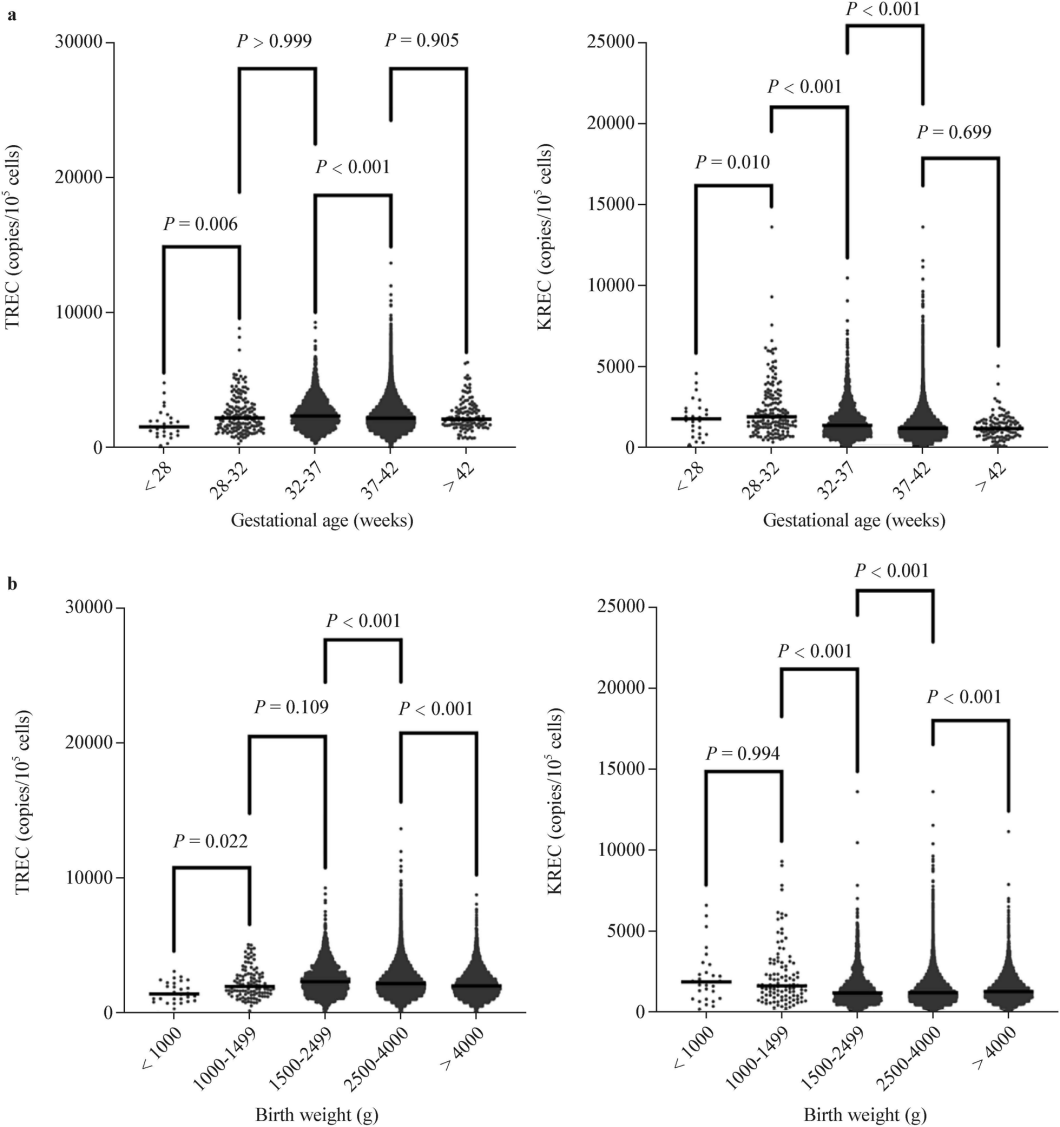
T-cell receptor excision circles (TRECs) and kappa-deleting recombination excision circles (KRECs) in newborns by **a** gestational age and **b** birth weight. *TREC* T-cell receptor excision circles, *KREC* kappa-deleting recombination excision circles

The screening diagnostic decision algorithm is illustrated in Fig. 1. Throughout the study period, comprehensive screening for SCID, XLA, and SMA was conducted on 103,240 newborns, covering 99.99% of all live births. Table 1 presents the baseline characteristics of the screened newborns, including sex, ethnicity, birth weight (BW), and gestational age (GA). The TREC and KREC values for these infants are shown in Fig. 2. The mean TREC levels were significantly lower in ultra-preterm infants (GA < 28 weeks) compared to other groups, with the highest values observed in very preterm infants (GA: 28–32 weeks). In contrast, the mean KREC levels peaked in very preterm infants and gradually declined with increasing GA, reaching their lowest in post-term infants (GA > 42 weeks). Similarly, the mean TREC levels were the lowest in very-low BW infants (BW < 1000 g) and highest in low BW infants (BW: 1500–2499 g). The mean KREC levels were highest in infants weighing 1000–1499 g, then rapidly declined with increasing weight, reaching their lowest in normal newborns (BW: 2500–4000 g).

**Table 1:** Baseline characteristics of the screened newborns

Characteristics	*n*	%
Gender		
Male	53,712	52.03
Female	49,528	47.97
Gestational age (GA)		
< 28 wk	27	0.03
≥ 28 wk, < 32 wk	162	0.16
≥ 32 wk, < 37 wk	2215	2.15
≥ 37 wk, < 42 wk	100,717	97.56
≥ 42 wk	119	0.12
Birth weight (BW), *g*		
< 1000	29	0.03
1000–1499	110	0.11
1500–2499	2077	2.01
2500–4000	95,507	92.51
> 4000	5517	5.34
Total	103,240	100.00

Among the newborns screened, 100,836 (97.67%) were born at full term, whereas 2,404 (2.33%) were delivered prematurely. A total of 699 samples with abnormal values and 7 incomplete samples from the initial analysis were subjected to repeated testing of the original DBS. After the retest, 122 samples (0.12%) had abnormal values: 40 with abnormal TREC, 72 with abnormal KREC, 1 with abnormalities in both TREC and KREC, and 9 with no amplification of *SMN1*. Of these, 117 were from full-term newborns (95.9%), while 5 were from premature newborns (4.1%). A total of 106 infants with abnormal TREC and/or KREC values underwent immunological testing, while the other 7 infants refused referral or were lost to follow-up. All nine infants who tested positive on SMA screening were referred for further evaluation. Overall, 115 of the 122 samples (94.26%) were successfully referred for the follow-up.

### Screening performance

The 106 referred infants with abnormal TREC/KREC values underwent a series of diagnostic immunological tests, including flow cytometry, immunoglobulin level assessments, and WES. In contrast, the seven unreferred infants were tested with WES on DBS samples as probands. MLPA was performed on all infants with undetectable *SMN1* cycle threshold (Ct) levels to assess the copy numbers of exons 7 and 8 in both *SMN1* and *SMN2*. We identified one patient with SCID, two patients with XLA, and nine patients with SMA, one of whom also had Wiskott**–**Aldrich Syndrome (WAS). In addition, eight patients were diagnosed with other medical conditions, such as trisomy 21 (*n* = 1), 22q11.2 deletion syndrome (*n* = 4), Noonan syndrome (*n* = 1), 47,XYY syndrome (*n* = 1), and pancytopenia (*n* = 1). At the time of publication of this article, no new cases had been identified among those with negative screening results. Therefore, the positive predictive values (PPVs) of NBS were 2.44% (1/41) for SCID, 2.78% (2/72) for XLA, and 2.65% (3/113) for PID. The specificities for SCID and XLA were 99.96% and 99.93%, respectively, with both conditions demonstrating a sensitivity and negative predictive value (NPV) of 100%. The PPV, NPV, sensitivity, and specificity for SMA were all 100%. Our overall screening performance for SCID, XLA, and SMA yielded a combined PPV of 9.84%.

### Confirmation and management

#### Primary immunodeficiency disorders

Table 2 presents comprehensive data on all individuals confirmed to have PIDs. Patient 1 (P1) exhibited abnormal TREC values in both the initial and repeat assays, while KREC values remained normal. Flow cytometric analysis of lymphocyte subsets revealed that the levels of CD3 + T cells (0.19×10^9^/L), CD4 + T cells (0.11×10^9^/L), CD19 + B cells (0.55×10^9^/L), and CD16 + CD56 + natural killer (NK)-cells (0.38×10^9^/L) were consistent with a T-B + NK + SCID phenotype. In addition, his serum immunoglobulin G (IgG) (5.0 g/L) and IgM (0.34 g/L) levels were within the normal ranges, whereas his serum IgA levels were low (0.01 g/L). Trio-WES identified a hemizygous mutation in intron 7 of the *IL2RG* gene (NM_000206.2: c.925-13 T > G) in both the patient and his mother. An identical mutation was detected in a DBS sample obtained from his deceased brother, who died from severe sepsis at 3 months of age. At 4 months old, P1 received a successful mismatched HSCT from his father. He is now nearly 2 years old and remains in excellent health.

**Table 2:** Summary of patients with SCID and XLA

Patients	Gender	GA (wk)	BW (g)	Age at DBS	TREC (copies/10^5^ cells)	KREC (copies/10^5^ cells)	Lymphocyte subsets (×10^9^cells/L)	Immunoglobulin (g/L)	Mutation genes	Diagnosis
P1	M	39^+6^	3830	3 d	0	293	Total 1.12 CD3 + 0.19 (17.6%) CD4 + 0.11 (10.15%) CD8 + 0.01 (1.25%) CD19 + 0.55 (49.15%) CD56 + 0.38 (34.05%)	IgG 5.0 IgA 0.01 IgM 0.34	*IL2RG*	T-B + NK + SCID
P2	M	40^+3^	3640	3 d	2963	0	Total 4.80 CD3 + 4.49 (93.6%) CD19 + 0.03 (0.7%)	IgG 7.2 IgA 0.01 IgM 0.02	*BTK*	XLA
P3	M	38^+4^	3100	4 d	1799	0	Total 5.04 CD3 + 4.60 (91.2%) CD19 + 0.03 (0.55%)	IgG 5.9 IgA 0.02 IgM 0.03	*BTK*	XLA

*M* male, *F* female, *GA* gestational age, *BW* birth weight, *DBS* dried blood spot, *TREC* T-cell receptor excision circles, *KREC* kappa-deleting recombination excision circles, *IgG* immunoglobulin G, *IgA* immunoglobulin A, *IgM* immunoglobulin M, *NK* natural killer cell, *SCID* severe combined immunodeficiency, *XLA* X-linked agammaglobulinemia

P2 and P3 initially exhibited an absence of KRECs in the DBS samples, which persisted in subsequent tests. Immunological evaluations revealed a B-cell deficiency (P2: 0.7% CD19 + B cells; P3: 0.55% CD19 + B cells) and hypogammaglobulinemia, including deficiencies in IgM and IgA (P2: IgM 0.02 g/L, IgA 0.01 g/L; P3: IgM 0.03 g/L, IgA 0.01 g/L), while IgG levels were normal (P2: IgG 7.2 g/L, P3: IgG 5.9 g/L) (Table 2). The XLA diagnosis for the two patients was confirmed by detecting maternal semi-zygotic variants in the *BTK* gene: c.92 T > C (p.L31P) in one patient and c.599dup (p.P201Afs*5) in the other. At 6 months of age, both P2 and P3 began receiving immunological replacement therapy, consisting of intravenous immunoglobulin infusions (400–600 mg/kg every 3–4 weeks). This treatment was necessary due to a reduction in IgG levels caused by the depletion of maternal IgG and a severe decrease in the endogenous production of IgG (P2: IgG = 0.9 g/L, P3: IgG = 0.6 g/L).

#### Spinal muscular atrophy

Cases P4 to P12, all of whom had positive SMA-NBS results, received a confirmatory diagnosis and determination of the *SMN2* copy number using MLPA. Among these patients, three had two copies of *SMN2*, while six had three copies, with no false-positive results observed.

P4, who had two copies of *SMN2*, was admitted to the newborn intensive care unit due to hypoxia at birth, along with hypotonia, joint contractures, and areflexia before the NBS results were available. Similarly, P5 and P6, who each had two copies of *SMN2*, exhibited hypotonia shortly after birth without any accompanying respiratory issues. Given the gravity of the illness, the projected outcome, and the financial burden of medical intervention, the families of P4, P5, and P6 opted to discontinue treatment, resulting in the tragic demise of all three infants within 2–6 months after birth.

At the time of diagnosis, none of the patients with three copies of *SMN2* (P7–P12) showed any symptoms. P7 through P10 began nusinersen treatment at a median age of 85 days (range: 59–137 days) and have remained asymptomatic, achieving age-appropriate milestones at their most recent follow-ups. P11, a migrant, was referred to a local hospital for follow-up care but did not receive treatment due to financial constraints. At her 6-month follow-up, P11 exhibited signs of delayed motor development and slight muscle weakness.

It is worth noting that P12, who had three copies of *SMN2*, was symptomatic at the time of nusinersen treatment. P12 was diagnosed with both SMA and WAS. On day 2 after birth, he developed petechial rashes and severe eczema all over his body, along with thrombocytopenia and the presence of small platelets. Trio-WES revealed a hemizygous nonsense mutation in exon 7 of the *WAS* gene (NM_000377.3: c.629C > G, p.Ser210Ter), inherited from his mother. Despite these findings, he had normal lymphocyte and immunoglobulin levels, consistent with the normal TREC/KREC values. P12 underwent an umbilical cord blood stem cell transplantation from a human leukocyte antigen-matched unrelated donor at 12 months of age, and nusinersen treatment was started at 15 months of age. Unfortunately, despite these interventions, P12 exhibited delays and regression in motor development since 6 months of age. Although subsequent evaluations showed slight improvements in motor development, his motor milestones remained significantly delayed. At his most recent visit, he had not yet achieved the ability to sit independently (Table 3).

**Table 3:** Summary of patients with SMA

Patients	Gender	GA (wk)	BW (g)	Age at DBS	*SMN1* Ct	SMN2 copies	Diagnosis	DMT	Age at treatment	Symptomatic at treatment	Age at last clinical	Symptomatic at last	Motor milestone achieved
P4	M	39^+3^	3500	3 d	NA	2	SMA	None	/	/	Died at 2 mon	Y	None
P5	M	31^+4^	1525	11 d	NA	2	SMA	None	/	/	Died at 6 mon	Y	None
P6	F	39^+2^	3270	3 d	NA	2	SMA	None	/	/	Died at 3 mon	Y	None
P7	F	38^+2^	3350	5 d	NA	3	SMA	Nusinersen	59 d	N	1 y 7 mon	N	Walking independently (11 mon)
P8	M	40	3170	3 d	NA	3	SMA	Nusinersen	137 d	N	1 y 6 mon	N	Walking independently (12 mon)
P9	M	38^+3^	2860	2 d	NA	3	SMA	Nusinersen	62 d	N	1 y 4 mon	N	Walking independently (13 mon)
P10	M	39^+3^	3610	3 d	NA	3	SMA	Nusinersen	82 d	N	1 y 4 mon	N	Walking independently (13 mon)
P11	M	40	3320	3 d	NA	3	SMA	None	/	/	6 mon	Y	Rolling; sitting with support
P12	M	39^+6^	3490	3 d	NA	3	SMA; WAS	Nusinersen	1 y 3 mon	N	2 y 3 mon	Y	Sitting with support

*NA* no amplification, *F* female, *M* male, *SMA* spinal muscular atrophy, *GA* gestational age, *BW* birth weight, *Ct* cycle threshold, *DMT* disease-modifying treatment, *Y* yes, *N* no, *WAS* Wiskott–Aldrich Syndrome, *NA* not available

#### Other medical conditions

Four patients diagnosed with 22q11.2 deletion syndrome (P13–P16) were identified through the NBS program due to their low TREC levels. P17, a patient with trisomy 21, was born as a 31^+4^-week premature twin and conceived through in vitro fertilization. P18 was diagnosed with Noonan syndrome after trio-WES revealed a de novo heterozygous variant of the *PTPN11* gene (NM_002834.5, c.922A > G). P19 initially presented with undetectable KREC copies, and further karyotyping confirmed a diagnosis of 47,XYY syndrome. Following referral, P19 was evaluated by a local immunologist, who found a normal immunophenotype along with normal lymphocyte and immunoglobulin levels. P20 was born with no measurable levels of TREC or KREC. Tragically, P20 died due to a combination of severe infection, respiratory failure, and pulmonary hemorrhage. Trio-WES did not reveal any genetic variations associated with immunodeficiency. Table 4 contains detailed information on all of these cases.

**Table 4:** Summary of other medical conditions

Patient	Gender	GA (wk)	BW (g)	Age at DBS	Diagnosis	TREC (copies/10^5^ cells)	KREC (copies/10^5^ cells)	Mutation genes
P13	M	41^+1^	2945	3 d	22q11.2 deletion syndrome	373	1463	2.68 Mb microdeletion in chromosome 22 long arm q11.2 (chr22:18,893,817–21,576,514×1)
P14	F	38^+3^	3250	7 d	22q11.2 deletion syndrome	287	185	2.92 Mb microdeletion in chromosome 22 long arm q11.2 (chr22:18,659,481–21,576,514×1)
P15	F	40	3450	4 d	22q11.2 deletion syndrome	60	1094	2.52 Mb microdeletion in chromosome 22 long arm q11.2 (chr22:18,893,817–21,414,846×1**)**
P16	F	39^+6^	3800	3 d	22q11.2 deletion syndrome	255	2988	2.52 Mb microdeletion in chromosome 22 long arm q11.2 (chr22:18,893,817–21,414,846×1)
P17	F	31^+4^	1470	3 d	Trisomy 21	1146	0	Chromosome 21 triploid repeat
P18	M	40	3700	3 d	Noonan syndrome	314	4054	De novo variation of PTPN11, c.922A > G (p.N308D), LP
P19	M	40	3400	7 d	47,XYY syndrome	5017	0	47, XYY
P20	F	38^+3^	3450	4 d	Pancytopenia	0	0	No definite pathogenic variation associated with phenotype was detected

*M* mail, *F* female, *GA* gestational age, *BW* birth weight, *DBS* dried blood spot, *TREC* T-cell receptor excision circles, *KREC* kappa-deleting recombination excision circles

In another case, the initial KREC score was slightly below the specified threshold. Subsequent investigations revealed that the infant’s mother had systemic lupus erythematosus (SLE) but was in remission after having undergone treatment. She was stable during pregnancy and did not receive any immunosuppressants. Immunological evaluation and follow-up reviews revealed a normal immunophenotype for the infant, and WES did not identify any gene variants associated with immunodeficiency. Consequently, this case is not included in Table 4.

## Discussion

Early screening, accurate diagnosis, and timely intervention for significant birth abnormalities are critical for improving disease prognosis and lowering medical expenses [25]. Both SCID and SMA are included in the recommended list of essential disorders for newborn screening by the Health Resources & Services Administration [21]. Currently, an increasing number of countries are either implementing or considering the inclusion of PID and SMA screening in their universal NBS programs.

In this study, the prevalence rates of SCID, XLA, and SMA in the Chinese population were estimated from the screening data. The occurrence rate of SCID was determined to be 1 in 103,240, which is significantly lower than that reported in Israel (1 in 22,159) [26], Wisconsin, USA (1 in 41,539) [27], and Taiwan of China (1 in 53,195) [28]. The reported incidence of XLA varies considerably by region and ethnicity: 1 in 200,000 live births in Switzerland [29]; 1 in 20,000,000 to 1 in 10,000,000 in Spain [30]; 1 in 285,000 to 1 in 100,000 in Norway [31]; 1 in 160,000 to 1 in 27,000 in Israel [32]; and 1 in 379,000 in the USA [33]. However, our study suggests that the prevalence may be higher in Chinese individuals, with an estimated rate of 1 in 51,620. Due to the relatively low occurrence of both SCID and XLA, it is necessary to obtain more accurate prevalence data by implementing generalized screening programs. The incidence of SMA in this study was 1 in 11,471, which is comparable to prevalence observed in other regions: Australia (1 in 10,390) [34], Taiwan, China (1 in 17,181) [35], and New York, USA (1 in 19,117) [36]. In addition, this figure is marginally lower than the prevalence reported in one of our previous multicenter studies (1 in 9,788) [37]. Owing to the extended screening period, larger sample size, and more consistent management of the single-center screening process across the entire region, the incidence rates reported in this study are likely to be more accurate.

Based on the current cut-off values for TREC and KREC, 113 samples (0.11%) below the cut-off value were considered abnormal TREC/KREC values. Among these patients, P20, who had pancytopenia, exhibited abnormal TREC and KREC values. Therefore, the positive rate for the TREC-alone assay was 0.03% (41/103,240), while the positive rate for the KREC-alone assay was 0.07% (73/103,240). This rate is comparable to figures reported in Sweden (0.10%; 93/89,462) [10] and Seville (0.097%; 5/5,160) [11], and is slightly higher than that reported in Japan (0.037%; 39/105,419) [12]. The rates reported here do not indicate a significant increase compared to other TREC-alone screening rates [38, 39]. In this study, we identified one patient with SCID and two patients with XLA, yielding a PPV of 2.44% for SCID and 2.78% for XLA. The overall PPV for PIDs was 2.65%. Considering the incidence of XLA observed in this study, a slight increase in the positivity rate for KREC is considered acceptable. However, higher cut-off values may lead to lower positive rates. While this approach could help reduce false positives, it may also risk overlooking cases of SCID or XLA, although the cases with undetectable TREC or KREC levels in our study still can be identified. In addition, it may also fail to identify other immunological disorders. Previous research has identified maternal immunosuppression, preterm birth [40], and congenital heart problems [41] as the primary factors contributing to T-cell and/or B-cell lymphopenia in infants. We found that TREC levels increased most rapidly when BW increased from < 1000 g to 1000–2499 g and GA increased from < 28 weeks to 28–32 weeks, corresponding to periods of rapid thymic development and increased T-cell production [42]. Conversely, the population median KREC levels decreased most rapidly as BW increased from 1000–1499 g to 1500–2499 g, and as GA progressed from 28–32 weeks to 32–37 weeks. This decline in KREC levels is attributed to B-lymphocyte production by the liver during the fetal period. As the liver rapidly increases in size and weight, active B-cell production occurs, diluting KREC concentrations in the peripheral blood [43, 44]. Analyzing the factors influencing these correlations can provide a theoretical basis for optimizing cut-off values, thereby ensuring sensitivity, reducing the false-positive rate, and improving screening efficiency.

Studies have shown that KREC analysis is valuable for classifying SCID and identifying B-cell deficiencies. KRECs can also be useful for detecting other PIDs, such as late-onset adenosine deaminase deficiency deficiencies, Nijmegen breakage syndrome, and other XLA-like disorders [14, 45]. While we did not detect other types of PIDs, our analysis revealed one case of SCID with undetectable TRECs (TREC; 0 copies/10^5^ cells) and two cases of XLA with undetectable KRECs (KREC; 0 copies/10^5^ cells). For instance, P1, who exhibited the T-B + NK + SCID phenotype, had abnormal TREC values and normal KREC values, which corroborates the role of KREC in SCID classification. In addition to SCID and XLA, our screening program identified eight patients with other medical conditions, including 22q11.2 deletion syndrome, Noonan syndrome, trisomy 21, 47,XYY syndrome, and pancytopenia of unknown etiology. 22q11.2 deletion syndrome, which is associated with thymic stromal deficiency leading to T-cell immunodeficiency, may sometimes exhibit low TREC levels while generally maintaining normal KREC levels [46]. In our study, all four patients with this condition presented with abnormal TREC and normal KREC values. Noonan syndrome, identified by abnormal TREC levels in our study, may be associated with a common phenotype of lymphatic dysplasia [47]. Thus, multiplexed TREC/KREC assays are both feasible and sensitive for the detection of T- and/or B-cell lymphopenia. The addition of KREC to our program did not affect the overall screening performance; moreover, it played a valuable role in predicting SCID subtypes. In addition, KREC screening is essential for the identification of trisomy 21 syndrome [48], and may also be valuable for detecting rare diseases such as 47,XYY syndrome [49].

Despite advances in genetic screening technologies and therapeutic methodologies that have accelerated the implementation of pilot SMA screening and led to its incorporation into routine neonatal screening programs in certain areas, global implementation remains limited. As of 2021, newborn screening for SMA has been implemented in only nine countries, representing less than 2% of the global newborn population [50]. Several pilot trials have expanded SMA-NBS by incorporating it into existing SCID-NBS panels. Our study not only addresses the lack of data in mainland China but also establishes the incorporation of measuring TREC/KREC/*SMN1* into our NBS program. Semi-quantitative detection of *SMN1* exon 7 by real-time PCR, used in NBS for SMA due to the homozygous deletion of exon 7, is a genetic screening method with a theoretical PPV of 100%. However, due to various constraints, including sample quality and primer design, the detection of exon 7 of the *SMN1* gene has been associated with a few false-positive results in prior investigations [34]. Our study did not identify any false positives, and *SMN1* Ct values were undetectable in all patients. This resulted in a PPV, sensitivity, and specificity of 100%, consistent with findings from most independent SMA studies, providing further evidence of the reliability and feasibility of our multiplex TREC/KREC/*SMN1* assays.

NBS provides a critical opportunity for pre-symptomatic diagnosis, allowing for early intervention and management of conditions. All three patients with PID in our study were promptly treated and closely followed up, including receiving genetic counseling. They were in excellent physical condition, had avoided recurrent infections, and experienced outcomes similar to those observed in P1’s brother. Previous studies have shown that children with SMA who begin treatment before symptoms appear have significantly higher survival rates, better respiratory and feeding support, and approved attainment of motor milestones compared to those who start treatment after the onset of symptoms [15]. In this study, the four children with three copies of *SMN2* who received nusinersen treatment before the onset of symptoms remained asymptomatic during the follow-up period, achieving age-appropriate motor milestones. Both untreated cases with three *SMN2* copies developed symptoms at 6 months of age and exhibited a tendency toward deterioration. P12 was a patient with combined SMA and WAS. During communication with the doctor, the parents were concerned that the subsequent HSCT would interfere with the effectiveness of the prior nusinersen treatment. Despite clear explanations from the medical team HSCT and nusinersen treatment could be administered concurrently without conflict, the parents insisted on prioritizing HSCT and postponing nusinersen treatment. P12 developed symptoms while waiting for a well-matched donor. Although his head control and muscle strength improved with symptomatic treatment using nusinersen, he remained incapable of sitting independently. We hypothesized that the clinical outcome of P12 was the result of multiple factors, including potential interference of WAS, implementation of HSCT, and the delayed initiation of nusinersen treatment. Despite the brief follow-up period and the potential influence of other medical conditions, the findings of this study align with prior SMA-NBS pilot research, demonstrating that children who received pre-symptomatic treatment through NBS showed substantial improvements in motor function [15, 36]. Unfortunately, all three infants with two *SMN2* copies discontinued treatment, resulting in inconclusive evidence regarding the impact of NBS on their prognosis in this study.

Previous studies have suggested that SCID-NBS is cost-effective [52–54]. While the cost-effectiveness of NBS for SMA has been disputed owing to the high cost of treatment drugs, comprehensive economic analyses from various nations indicate that NBS for SMA is both more cost-effective and clinically beneficial, compared to scenarios without screening [55, 56]. Adding SMA-NBS to SCID-NBS incurs only a minimal additional cost when using a multiplex qPCR assay, which further reduces the overall cost of SMA screening. Economic analysis of NBS programs in Australia for SCID and SMA demonstrates significant health and financial benefits [57]. The combined screening approach is projected to yield 95 quality-adjusted life-years (QALYs) per 100,000 newborns screened, while simultaneously generating cost savings of $8.6 million. These data strongly support the cost-effectiveness of implementing a joint NBS program for SCID and SMA [51]. Tesorero et al. [58] integrated sickle cell disease screening into the original NBS panel, suggesting that a multiplex real-time PCR assay could be integrated with the current screening tests to include additional target diseases through the analysis of disease-associated DNA molecules or genes. In the present study, we used a multiplex real-time PCR assay to minimize the cost of NBS for SCID, SMA, and XLA. Given that disease incidence significantly impacts cost-effectiveness, the higher prevalence of XLA in the Chinese population identified in our study underscores the significance and potential cost of implementing NBS for this condition. The timely interventions made possible by our screening significantly improved the prognosis of affected infants. As the main factor in the cost-effectiveness of SMA-NBS, the recent reduction in the price of nusinersen in mainland China to 33,000 RMB has considerably lowered overall treatment costs. However, related studies have not yet been performed in China. Considering disease incidence, the reliability of screening techniques, treatment accessibility, and improved prognosis, we anticipate that combined TREC/KREC/*SMN1* screening will not only provide long-term cost savings for the government but will also improve and save lives.

Despite limitations such as reduced specificity, the need for additional validation, and the potential for false-positive or false-negative results, molecular screening using multiplex real-time PCR assays offers a convenient and cost-effective complementary approach to traditional biochemical screening in public health NBS programs. WES is crucial for identifying children with other genetic diseases, such as 22q11.2 deletion syndrome and atypical biochemical markers. Recent advancements in genetic testing technology have led to increased attention on applying next-generation sequencing (NGS) to NBS. NGS offers significant advantages, including high-throughput capabilities and shortened diagnostic times for patients with complex medical conditions [59]. In our study, a subset of 26 neonates with abnormal TREC/KREC screening results were found to carry likely pathogenic mutations as identified by WES. However, because these patients did not exhibit phenotypic confirmation at the time of referral, they were excluded from the list of confirmed patients. These cases continue to be monitored, and this issue will be addressed in subsequent follow-ups. Therefore, employing NGS as a first-tier test for NBS could significantly increase the costs of both screening and confirmatory testing and increase the requirements for gene interpretation, genetic counseling, and long-term management [60, 61]. Building on insights from our previous multicenter study [62], we have begun implementing NGS-NBS as an optional component in our NBS program in Zhejiang Province. We believe that biochemical combined genetic screening represents a promising future trend in NBS.

In summary, this study employed real-time PCR to concurrently screen for SCID, SMA, and XLA in a single assay, demonstrating the clinical effectiveness of this combined screening approach in a large cohort of newborns. In addition to validating the diagnoses of the associated diseases and identifying patients with specific syndromes, we developed an integrated system for early neonatal screening, diagnosis, treatment, and follow-up that is ready for widespread adoption and implementation. This system encompasses the creation of a comprehensive epidemiological database in mainland China, the establishment of a disease cohort, the integration of multi-disciplinary team treatments, and the standardization of clinical pathways. Negative screening results should also be managed through comprehensive pediatric healthcare services established in China [63], which provide continuous healthcare services for all children from birth. For asymptomatic children, genetic counseling and health management can be offered based on genetic analysis and genotype–phenotype correlations. The establishment of a standardized screening system is expected to increase the utility of NBS.

## Data Availability

The datasets generated during and/or analyzed during the current study are available from the corresponding author on reasonable request.
